# Post-Publication Peer Review: Opening Up Scientific Conversation

**DOI:** 10.3389/fncom.2012.00063

**Published:** 2012-08-30

**Authors:** Jane Hunter

**Affiliations:** Faculty of 1000, Ltd.London, UK

## Conventional Peer Review: Rights and Wrongs

Peer review is broken. We have all heard that phrase many times in recent years. It’s become a truism, a shorthand complaint about the *status quo* that rarely extends into a proposal for change. And even those who do not believe standard peer review is beyond repair acknowledge that there are problems; everyone can see the cracks.

So what’s wrong? From an author’s point of view, a lot. Peer review is slow; it delays publication. It’s almost always secret; authors do not know who is reviewing their work – perhaps an ally but, equally, perhaps a competitor. It can block ingenuity; think of the classic case of Lynn Margulis and the 15 or so journals that rejected her ground-breaking article “On the origin of mitosing cells” (Sagan, 1967) before it was finally accepted by *The Journal of Theoretical Biology*. And there’s a lot wrong for reviewers too: what proportion of referee reports are second, third, or even fourth round reviews? A referee’s hard work may be contributing nothing new to an author who would rather take his or her chances with another journal than do the extra work suggested by reviewers for journals one to three.

Does conventional peer review work for publishers? Well, yes and no. Yes, at top-flight journals like *Nature* or *NEJM* peer review is a gate keeper that helps guarantee publication of only the most interesting articles, and yes, in theory, it helps guard against the publication of flawed work, but it’s expensive – even though reviewers work for free – and it’s time-consuming. *Nature* or *NEJM* review thousands of papers each year that would not make it into their journals; for third-, fourth-, or fifth-tier journals, somewhere further down the inevitable cascade, referees will often be doing work that has been done already on an article that was written months ago.

If standard peer review is intended to help ensure that an article is good enough to be published, is it working? And in this context, what does “good enough” even mean? Since most papers will eventually be published, cascading until they find a journal, that means that most papers are good enough for someone and peer review’s supposed qualitative gatekeeper role is not supportable. The impact of peer review on the publication of an article is not so much a question of yes or no, it’s more likely to be a question of when and where.

Yet even acknowledging the flaws, redundancies, and costs of the conventional peer review system, it is clear that we need peer review. The more specialized science becomes the more we must rely on experts to help us navigate the multiplicity of subject areas we are not expert in ourselves. Peer reviewers are those experts and we depend on the refereeing process to protect us from sloppy work and invalid conclusions.

So peer review is important but the way it happens is problematic

At F1000, we believe that most of the weaknesses of standard peer review can be linked to two core issues, first that it is conducted pre-publication and second that it is secret. Pre-publication peer review allows journals and reviewers to delay, filter, and interrupt the essential conversation of science, and secrecy makes these problems impossible to resolve.

## Post-Publication Peer Review: Two Models from Faculty Of 1000

A little background: faculty of 1000 began in 2002 with a post-publication review service called F1000 Biology. Its remit was (and still is) to work with named experts to identify and recommend the most interesting papers published across 24 different subject areas in biology. In 2006 F1000 Medicine joined it – with the same aim, more experts and coverage of 20 medical specialties. We merged the two services in 2010, and biology and medicine are now both covered at F1000.com.

Since then, we have launched F1000 Posters, an open access repository for posters and presentations – again in biology and medicine – and we are now in the early stages of launching our new open access, post-publication peer review journal, *F1000 Research*.

Faculty of 1000 practices two forms of post-publication peer review: primary, open refereeing of articles after they are published in *F1000 Research*, and secondary peer review of the best already-refereed articles, published in any biology or medicine journal, at F1000.com. Both are illustrations of Clay Shirky’s “publish then filter” model (Shirky, 2008) and each adds value to scientific discourse in its own way.

I will describe our secondary post-publication review process first.

## Secondary Post-Publication Peer Review

The F1000 article recommendation service applies a layer of positive filtering on top of traditionally peer reviewed literature; we review already-published biology and medicine in order to identify and promote the best work. Our 10,000 named Faculty Members and their Associates select articles that impress them, regardless of source, and write brief recommendations explaining what makes the work significant and putting the science in perspective. These recommendations and comments, along with links to the original articles, are published on F1000.com.

Why is this a useful thing to do? It’s useful because the vast volume of material published each year (or each day) makes it difficult for researchers to stay up to date with their own specialized fields, let alone with peripheral fields – all those other subject areas they should be keeping an eye on. Sure, you can search for articles and find, more or less, what you are looking for, but it’s helpful to have access to expert opinion for timely guidance on what’s especially significant and why. The fact that F1000’s reviewers are named puts their opinions in perspective. No one has ever suggested that our F1000 Faculty Members should conduct this form of post-publication review anonymously.

## Primary Post-Publication Peer Review

*F1000 Research*, F1000’s new primary open access publishing program in biology and medicine, publishes immediately, and offers fully open, post-publication peer review. We published our first articles in mid-July and are planning for a full launch at the end of this year.

Articles submitted to *F1000 Research* are first processed through an in-house sanity check and then, assuming they pass, published immediately. Post-publication they are subjected to formal peer review. Referees’ reports are published on the site and all referees are named.

The most important task for our referees is to tell us immediately whether or not an article is good science. We do not need to know if it’s exciting, or novel, or ground-breaking, we simply want to know that it’s valid; that it’s sensible work, carefully done. We expect the vast majority of submissions to be approved as good science. If it is good science, an article will be marked as such. If it’s not, or if it’s good science but the referee has reservations, we require that the referee add a report describing the problems and – if applicable – suggesting improvements. We encourage, but do not require, referees to add reports to articles they have approved as good science.

Authors have the opportunity to respond to a referee’s comments and are encouraged to update their articles and publish revised versions on the site. All versions are separately citable. All articles and all versions are clearly marked with their referee status and articles that have not yet been refereed are labeled as “Awaiting Review.”

The strengths of this model are that it’s fast, all good science can be published immediately and become part of the record to the benefit of scientists and others worldwide; it’s fair, publication cannot be blocked or slowed by the refereeing process; and it’s open, and openness discourages bias.

We do not see many weaknesses or risks with this model ourselves – standard peer review has few fans and is overdue for change – but then you might expect us to say that. We do understand though that there are concerns. These include:

–*Is there a risk that F1000 Research will publish junk?*: No, there is not. It will publish good science and let the community decide what the ultimate value of a specific piece of work is. As an aside, we expect that less junk – however one might define that term in science – will be submitted to *F1000 Research* than to conventional journals because few people will want to see a severely negative review of their work become part of the public record. Because *F1000 Research* will publish immediately then review openly, sloppy work will be publicly described as such.
–*OK, if not junk then uninteresting science*: Maybe, maybe not. Uninteresting science is still science, and we believe it should be published. There is a reason for top-line journals to sharply restrict what they publish, that’s how they create and maintain their identities and Impact Factors, but it’s hard to argue that such restrictions on scientific discourse are, overall, a good thing. We believe they are not. Valid science should be published.
–*No reviewer will want to be openly negative about another scientist’s work*: Having now published our first articles we are seeing in real time that this is not the case. Referees are happy to criticize and authors are happy to be able to respond, to present their case. And because everything is happening in the open, interested scientists can, for the first time, read the back-and-forth and make up their own minds.

*F1000 Research’s* version of “publish then filter” is an innovation in life-science publishing and no doubt additional concerns will arise as we fine-tune our model. However, it’s clear to us that the research community as a whole is more than ready to contemplate and, we believe, support real change. Complaints about conventional, pre-publication, closed peer review systems are mounting and the risks associated with our “publish first/referee openly later” system seem relatively trivial when compared with the increasing expense and frustration associated with the *status quo*.

We were the inventors of and original advocates for open access. We created Biomed Central, helped set up PubMed Central, and fought the publishing establishment for years to prove that open access can work, that it can be a profitable alternative to standard subscription models. *F1000 Research* and its novel publishing model take openness to the next level. Open access removes barriers for readers. Open, post-publication refereeing removes barriers for readers and authors alike, and it refocuses the role of peer review from, at its worst, a behind-the-scenes variety of censorship to, at its best, the process of expert criticism and advice that has always been its core and upon which the progress of science depends.
